# Long-Term Social Isolation-Induced Autophagy Inhibition and Cell Senescence Aggravate Cognitive Impairment in D(+)Galactose-Treated Male Mice

**DOI:** 10.3389/fnagi.2022.777700

**Published:** 2022-03-24

**Authors:** Bin Wang, Michael Ntim, Min Xia, Ying Wang, Jin-cheng Lu, Jin-Yi Yang, Shao Li

**Affiliations:** ^1^Department of Physiology, College of Basic Medical Sciences, Liaoning Provincial Key Laboratory of Cerebral Diseases, National-Local Joint Engineering Research Center for Drug-Research and Development (R&D) of Neurodegenerative Diseases, Dalian Medical University, Dalian, China; ^2^Department of Physiology, School of Medicine and Dentistry, Kwame Nkrumah University of Science and Technology, Kumasi, Ghana; ^3^Department of Cardiology, Institute of Heart and Vessel Diseases of Dalian Medical University, The Second Affiliated Hospital of Dalian Medical University, Dalian, China; ^4^Department of Urology, Affiliated Dalian Friendship Hospital of Dalian Medical University, Dalian, China

**Keywords:** aging, long-term post-weaned social isolation, cognition, memory, D(+)Galactose, autophagy, cell cycle

## Abstract

Aging is associated with physiological and pathological changes and presents health complications, such as dementia. Isolation has also been associated with the experience of growing old. Both have been linked individually to the incidence of cognitive decline. In this present study, the effects of these two phenomena have been looked at in animal models where aging was induced with D(+)Galactose in mice who underwent long-term post-weaned social isolation (L-PWSI). Assessing cognitive function using Y-maze, Morris water maze (MWM), and passive avoidance tests (PATs) confirmed that cognition is impaired in either of the treatments but worsened when the D(+)Galactose mice were subjected to L-PWSI. Moreover, a synaptic protein, PSD95, and dendritic spines density were significantly reduced in the L-PWSI and D(+)Galactose-treated mice. Our previous study revealed that autophagy deficit is involved in cognitive impairment in the L-PWSI model. Here, we first report the inhibited cell cycle in L-PWSI, combined with the decreased autophagy, aggravates cognitive impairment in D(+)Galactose-treated mice. Beyond these, the autophagy and cell cycle mechanisms that link isolation and aging have been explored. The close association between isolation and aging in humans is very real and needs much research attention going forward for possible therapeutic interventions.

## Introduction

Aging, an inevitable change that is experienced by all living organisms, is associated with some physiological and pathological processes that end with health complications and diseases. Some of these diseases include cardiovascular diseases, neurodegenerative diseases, and cancers (Dillin et al., 2014). Globally, it is expected that the aging population will increase and could pose a huge social burden. Reversing aging has gained much attention in biomedical research which has warranted more aging studies to understand the processes involved (Kim et al., 2018). Undoubtedly, aging has been seen as a biological process that features a progressive degeneration of physiological functions, and this results in high morbidity and death rate. Aging is one of the main contributors to cognitive declines, impairments in learning and memory, and the onset of dementia (such as Alzheimer’s disease) (Morrison and Hof, 1997; Raz et al., 2010). The effect of aging is at its highest when humans reach about 90 years (in both men and women) (Meng et al., 2010).

Loneliness and isolation have been seen as part of the experience of growing old. Reduced intergenerational living, the rise in one-person households, and many other factors predict that older adults may become more socially isolated (Valtorta and Hanratty, 2012). Cognitive processes have been implicated as a potential that links loneliness and health (Ong et al., 2016). Social isolation is an objective reflection of reduced social network size or lack of social contact and this has been linked to damage in the brain and could ultimately result in cognitive impairments (Yusufishaq and Rosenkranz, 2013).

Linking the effect of aging and social isolation to cognitive decline has become very critical in developing therapies that can attenuate both phenomena that are common at a point in one’s development. Using the much-accepted model for aging in mice, this study employed the administration of D(+)Galactose to induce aging in mice (Shwe et al., 2020; Hong et al., 2021) and assessed the effect of both aging and social isolation on cognition. Autophagy, a process utilized by cells to maintain homeostasis is very critical in cognitive performance (Dikic and Elazar, 2018). To some extent, the autophagy reactivation restored the expansion of geriatric cells and prevented senescence, as shown in reduced cell senescence maker (García-Prat et al., 2016; Bi et al., 2018). Hence, cell senescence makers and autophagy markers were studied to look at the effectiveness of establishing the aging condition in this model. This study provides preliminary evidence to suggest that a combination of aging and social isolation significantly worsen cognition than either of the two alone and suggests that long-term post-weaned social isolation (L-PWSI)-induced autophagy and cell cycle inhibition could be involved in this mechanism.

## Materials and Methods

### Animals and Habituation

Male Balb/c mice were purchased from the Laboratory Center of Dalian Medical University. The mice were assigned to group housing versus isolation housing with free access to food (mouse chow) and water. The animals were housed in polypropylene cages with woodchip bedding, and the housing rooms were set to a 12-h day/night cycle at 21 ± 1°C and 55 ± 5% humidity. For the L-PWSI model, the mice were separated and individually housed in cages from the first weaning day, which is postnatal day 21, and the isolation period lasted for approximately 15 weeks. The isolated mice only had auditory and olfactory contact with other conspecifics without any form of physical interaction or visual contact with the other conspecifics. After 8 weeks of isolation, aging was induced in mice by administering D(+)Galactose (BS917-25g, Biosharp) in normal saline once daily at a dose of 150 mg/kg *via* intraperitoneally (i.p.) injection for 8 weeks as Nagarajan et al., with slightly modified (Azman and Zakaria, 2019; Maharajan and Cho, 2021), the corresponding control groups received equivalent normal saline. This was done while isolation was ongoing.

**FIGURE 1:**
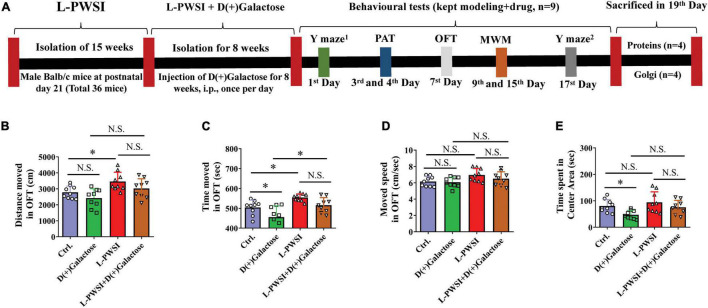
Experimental timelines and open field test (OFT) results to reflex the exploratory activities and anxious behaviors. **(A)** Experimental timelines. The long-term post-weaned social isolation (L-PWSI) mice were individually housed from postnatal day 21 for 15 weeks, and the control mice were group housing. In the course of modeling the L-PWSI (8 weeks), mice were injected with D(+)Galactose till the end of 15 weeks of L-PWSI. After the modeling, the animals underwent weeks of behavioral testing (the L-PWSI mice were still isolated) i.e., Morris Water Maze, Y-maze, and Passive avoidance tests. The mice were allowed to rest every other day during the behavior testing. At the end of the experiment, animals were sacrificed and samples were collected for western-blot and Golgi. The results of the OFT show anxiety measures. **(B)** Means of distance moved, **(C)** time moved, **(D)** movement speed, and **(E)** the time spent in the center zone in the OFT. Data are presented as the mean ± SEM from nine mice in each group. * represents *p* < 0.05; N.S., represents no significance.

The mice were allowed an adaptation period of 7 days in the behavioral experiments room with free access to food and water. Mice were grouped into four, i.e., Control (without isolation or D(+)Galactose treatment), L-PWSI, D(+)Galactose-treated, and L-PWSI + D(+)Galactose-treated groups. The number of mice in each group and tested in the behavioral experiments and the timelines have been represented in detail in Figure 1A. The behavior experiments were done immediately after the model was completed.

### Behavioral Tests

All the behavioral tests were performed by the EthoVision XT system (Noldus, Netherlands). The Morris water maze (MWM) was performed as described in our previous studies (Jiang et al., 2020).

#### Open Field Test

To assess the basic locomotor activity and anxiety-like behavior, the OFT was performed. The activity state of mice in the open field area (50 cm × 50 cm × 40 cm) was recorded for 10 min. The total distance moved, moved time, moved speed, and time spent in the center zone (15 cm × 15 cm) of the field were calculated.

#### Morris Water Maze Test

In the MWM test, the acquisition phase consisted of 5 consecutive trial days (3 trials per day), the memory consolidation test was administered 24 h later without the platform (two trials). The relative escape latency was calculated by normalizing the escape latency of the first day to 1.0 and calculated the relative escape latency for the subsequent trial days to those from the first day, and the time spent in targeted quadrant ratio, cumulative distance, and swim speeds.

#### Passive Avoidance Test

Passive avoidance learning was performed using a shuttle box, comprised of a brightly illuminated compartment connected to a dark compartment *via* a controllable door (Jiang et al., 2020). The latency, frequency, and permanence time to (in) the dark compartment are the parameters for cognitive activity determination. Increased latency, decreased frequency, and permanence time to (in) the dark compartment on the retention trial (second day) as compared to the acquisition trial (first day) indicate better learning ability. For the acquisition trial on the first day: each mouse was first placed in the bright compartment for 3-min acclimatization, the mouse could randomly move across the compartments. After that, once the mouse reached the bright compartment, the mouse was subjected to a trial of 5 min. Upon complete entry into the dark compartment during the trial period, the mouse received a slight foot electric shock through the floor grid (0.2 mA for 2 s). The latency time to re-enter the dark compartment, the frequency, and permanence time in the dark compartment were recorded. For the retention trial on the second day, the mice were tested for 5 min without acclimatization. The latency, frequency, and permanence time to (in) the dark compartment were recorded.

#### Y-Maze Task

The spontaneous alternations of mice in the Y-maze refer to the natural tendency of mice to spontaneously choose alternate arms, a quick simple test of spatial memory (Peng et al., 2016; Gervasi et al., 2018). The Y-maze apparatus was made of three opaque identical plastic arms (7.5 × 15 × 30 cm, labeled as A, B, and C) placed at an angle of 120° to each other. The mice were first put in the center of the maze and allowed to freely explore the three arms for 5 min each. Four limbs into one arm of the Y-maze was defined as the arm entry. Entry into three different arms in succession was defined as one alternation (e.g., ABC, CBA, BCA, or CAB arms). The total alternations, novel alternations, and the novel alternation preference ratio were tracked and analyzed using the EthoVision software (Noldus Information Technology Inc., Netherlands). The novel alternation percent score was calculated using the following equation: novel alternation (%) = [(number of alterations)/(total arm entries - 2)] × 100%. Y-maze new arm test was performed to detect the episodic memory, which was separated into acquisition (one arm was closed as the novel arm for 5 min), consolidation (mouse was put back into the living cages for 2 h), and retrieval (three arms are open for 5 min) parts. The mouse was first habituated in the acquisition part and 2 h consolidation and put back into the maze once again to register memory retrieval.

### Western Blot and the Methods to Dissect the Hippocampus

The western blot experiments and the methods to dissect the hippocampus were carried out according to our previously described method (Wang et al., 2019). The primary antibodies: anti-β-actin (ab6276, Abcam, United States), anti-PSD-95 (ab2723, Abcam), anti-p62/SQSTM1 (P0067, Sigma-Aldrich), anti-p16/INK4A (10883-1-AP, ProteinTech), anti-cyclin D1 (BM0771, Boster), anti-p-ULK (Ser757, 14202T, Cell Signaling Technology), anti-ULK (8054S, Cell Signaling Technology), anti-mTOR (2983S, Cell Signaling Technology), anti-phosphorylated mammalian target of rapamycin (p-mTOR; Ser2448, 5536S, Cell Signaling Technology), anti-PCNA (10205-2-AP, ProteinTech), anti-HO-1 (ab13248, Abcam), anti-SOD1 (37385, Cell Signaling Technology), and anti-Nrf2 (ab62352, Abcam).

### Golgi Staining for Dendritic Spines

The dendritic spines on the secondary and tertiary branches of pyramidal neurons in the hippocampus were observed by Golgi-Cox staining according to our previously described method (Jiang et al., 2020). The number of apical spines on hippocampus neurons was counted from 10 photographs per mouse in the digitized images.

### Statistical Analysis

**FIGURE 2:**
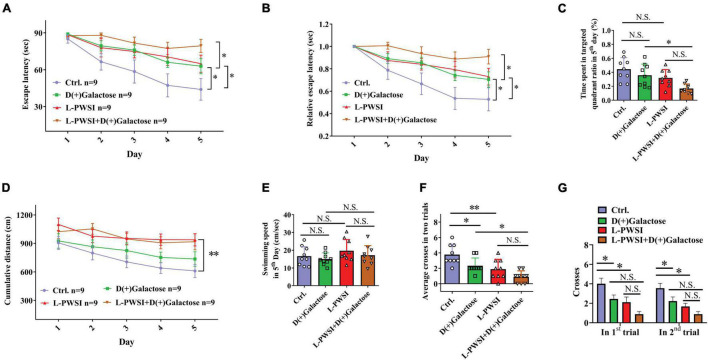
Spatial learning behavior is worsened in the long-term post-weaned social isolation (L-PWSI) and D(+)Galactose-treated mice in the Morris water maze (MWM). **(A,B)** The escape latencies and relative escape latencies were calculated for the various treatment and control groups of mice. The escape latencies of the four groups of mice on the first day were normalized to 1.0. The relative escape latencies on the subsequent days were calculated relative to those on the first day. **(C)** The total time spent in the targeted quadrant on the fifth day of the trial. **(D)** The cumulative distance (to the platform) during the training session (acquisition session). **(E)** The swimming speed of mice on the fifth day of the trial. **(F)** The average number of times that the four groups of mice swam across the target sites after retrieval of the platform (two trails). **(G)** The number of crossing the target sites in each trail after retrieval of the platform. Data are presented as the mean ± SEM. from 9 mice in each group. * represents *p* < 0.05; ** represents *p* < 0.01; N.S., represents no significance.

**FIGURE 3:**
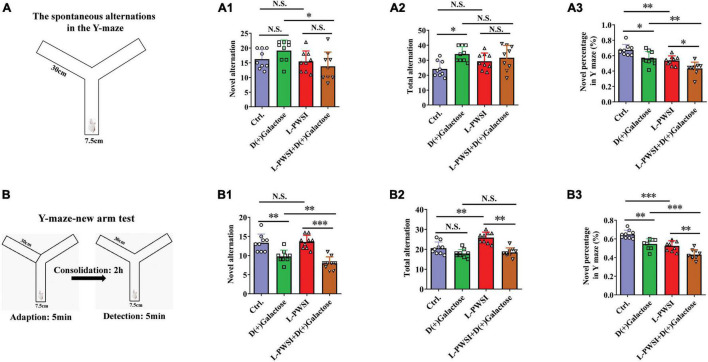
The results of the Y-maze-spontaneous alternation test and New arm test in the D(+)Galactose, long-term post-weaned social isolation (L-PWSI), L-PWSI + D(+) Galactose and control groups of mice. **(A)** Schematic representation of the Y-maze-spontaneous alternation test. Means of novel alternation **(A1)**, total alternation **(A2)**, and novel percentage **(A3)** in Spontaneous alternation test. **(B)** Schematic representation of the Y-maze new arm test in the consolidation memory test. Means of novel alternation **(B1)**, total alternation **(B2)**, and novel percentage **(B3)** in New arm test. Data are presented as the mean ± SEM. from 9 mice in each group. * represents *p* < 0.05; ** represents *p* < 0.01; *** represents *p* < 0.001; N.S., represents no significance.

**FIGURE 4:**
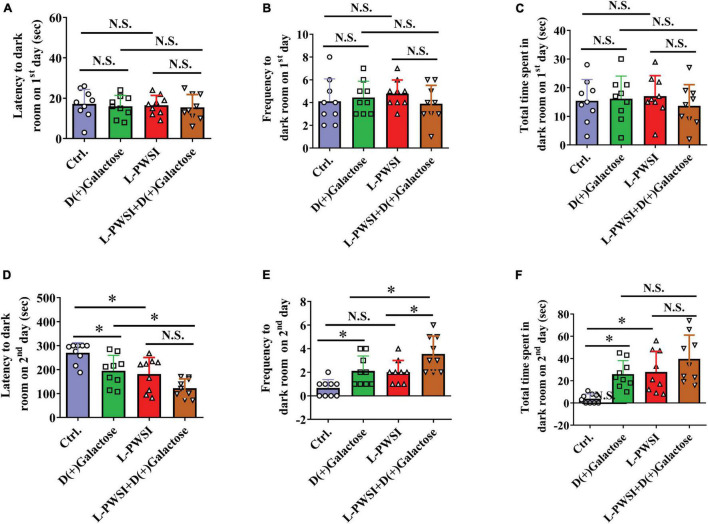
Treatment with long-term post-weaned social isolation (L-PWSI) and D(+)Galactose affected escape learning behavior in PAT. The latency **(A)**, frequency **(B)**, and permanence time **(C)** to (in) the darkroom on the first day of PAT. The latency **(D)**, frequency **(E)**, and permanence time **(F)** to (in) the darkroom on the second day of PAT. Data are presented as the mean ± SEM. from 9 mice in each group. * represents *p* < 0.05; N.S., represents no significance.

**FIGURE 5:**
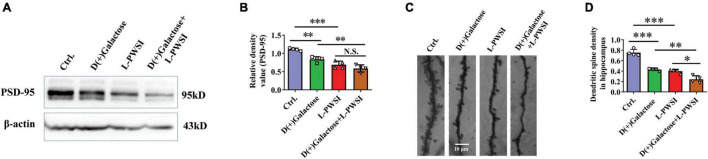
Treatment with long-term post-weaned social isolation (L-PWSI) and D(+)Galactose decreased PSD-95 and dendritic spine density. Representative micrographs **(A)** of Western blot (protein expression) with densitometry analysis PSD-95 **(B)** in the hippocampus. The grouping of gels/blots is cropped from different parts of the same gel. **(C)** Representative micrographs of dendrites **(D)** and dendritic spines density in the hippocampus. Each data column represents the mean ± SEM obtained from 4 brain samples. * represents *p* < 0.05; ** represents *p* < 0.01; *** represents *p* < 0.001; N.S., represents no significance.

**FIGURE 6:**
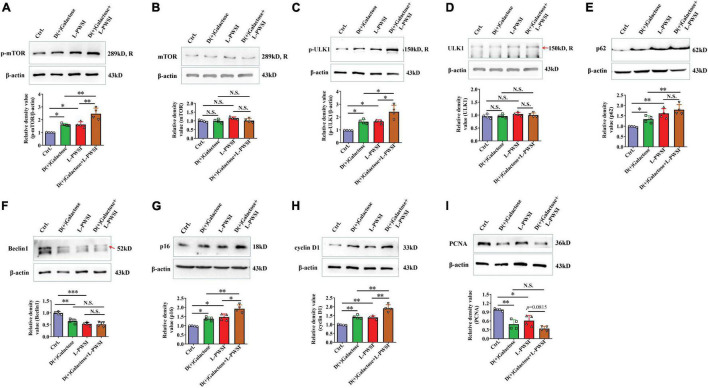
Treatment with long-term post-weaned social isolation (L-PWSI) and D(+)Galactose altered autophagy and cell-cycle molecules. Representative micrographs of Western blot (protein expression) with densitometry analysis of **(A)** phosphorylated mammalian target of rapamycin (p-mTOR), **(B)** mTOR, **(C)** p-ULK1, **(D)** ULK1, **(E)** p62, **(F)** Beclin 1, **(G)** p16, **(H)** cyclin D1, and **(I)** PCNA in the hippocampus. The grouping of gels/blots is cropped from different parts of the same gel. Each data column represents the mean ± SEM obtained from four brain samples. * represents *p* < 0.05; ** represents *p* < 0.01; *** represents *p* < 0.001; N.S., represents no significance.

The data were analyzed using GraphPad Prism (GraphPad Software Inc.), expressed as the mean ± SEM. Two-way ANOVA was used among these four groups (Figures 1, 2C,E–G, 3–6), Tukey’s multiple comparisons test was used for the comparison between two groups. The escape latency and relative escape latency data were assessed by repeated-measures ANOVA, as shown in Figures 2A,B,D. *p* < 0.05 was considered statistically significant.

## Results

### Spatial Learning Memory Is Worse in Long-Term Post-weaned Social Isolation and D(+)Galactose-Treated Mice

Prior to testing spatial learning, anxiety measures were determined by subjecting the mice through an OFT. Compared with the control, the L-PWSI group showed increased distance moved [*F*_(3,24)_ = 7.361, *p* < 0.01, Figure 1B]; the D(+)Galactose group showed anxiety-like behavior as decreased time spent in the central zone [*F*_(3,24)_ = 6.603, *p* < 0.01, Figure 1E]; the L-PWSI group showed increased total time moved, which was decreased in D(+)Galactose group [*F*_(3,24)_ = 10.89, *p* < 0.001, Figure 1C]; no significant difference in moved speed [*F*_(3,24)_ = 2.846, *p* = 0.0588, Figure 1D].

Morris water maze test was used to measure spatial learning and memory function (Vorhees and Williams, 2006). The repeated-measures ANOVA demonstrated a significant difference between-subjects effects (for time) in the escape latencies [*F*_(1,32)_ = 15.625, *p* < 0.01] (Figure 2A), suggesting intact learning capabilities of these mice. Repeated-measures ANOVA showed a significant difference between-subjects effects (for group) in the escape latencies [*F*_(3,32)_ = 5.239, *p* < 0.01] (Figure 2A), suggesting a significance among these groups. The control group compared with the L-PWSI group or D(+)Galactose group and the D(+)Galactose group compared with the L-PWSI and D(+)Galactose-treated group, all showed impaired or worsened learning in the activity of locating the hidden escape platform, which was depicted by the increased escape latencies during the trials on the fifth day (*p* < 0.05). The repeated-measures ANOVA showed no significant difference within-subjects effects (time × group) [*F*_(3,32)_ = 2.789, *p* = 0.056] (Figure 2A).

Similar results with the escape latencies, the L-PWSI and D(+)Galactose-treated mice exhibited significantly worse relative escape latency on the fifth day when compared to that of the D(+)Galactose-treated mice [repeated-measures ANOVA between-subjects effects (for group): *F*_(3,32)_ = 5.672, *p* < 0.01; within-subjects effects (time × group): *F*_(3,32)_ = 3.303, *p* < 0.05, Figure 2B].

The time spent in the target quadrant on the fifth day was also determined. No significant change was found in the D(+)Galactose or L-PWSI group compared with the control. Compared with the D(+)Galactose, the L-PWSI and D(+)Galactose-combined treatment groups showed a significant decrease in the time spent in the target quadrat on the fifth day [*F*_(3,24)_ = 5.729, *p* < 0.01, Figure 2C]. With regards to the cumulative distance (acquisition session) to the platform (Figure 2D), the repeated-measures ANOVA demonstrated a significant difference between-subjects effects (for time) [*F*_(4,128)_ = 5.520, *p* < 0.001] (Figure 2D) and between-subjects effects (for group) [*F*_(3,32)_ = 11.627, *p* < 0.001] (Figure 2D) in the cumulative distance. There was no significant difference within-subject (time × group) for distance [*F*_(12,128)_ = 0.316, *p* = 0.985] (Figure 2D). These results are similar to the escape latency. No significant differences were observed in the swimming speed among all the groups [*F*_(3,24)_ = 1.166, *p* = 0.3435, Figure 2E] indicating that all the groups had equal ability to swim. In memory consolidation assessments, compared to the control group, the L-PWSI mice and the D(+)Galactose-treated group showed a decreased average number of times the mice swam across the platform zone (when the platform was removed) [*F*_(3,24)_ = 10.33, *p* < 0.01, Figure 2F]. The case was significantly worse in the L-PWSI and D(+)Galactose-treated group (*p* < 0.05) compared with the D(+)Galactose group. Specifically, the number of crosses in each trial was analyzed and found a significant decrease in the L-PWSI only and D(+)Galactose only-treated groups compared with the control in the first trial [*F*_(3,24)_ = 7.393, *p* < 0.01, Figure 2G] and the second trial [*F*_(3,24)_ = 9.680, *p* < 0.001, Figure 2G], whereas no significance between the L-PWSI only or D(+)Galactose only-treated groups and the double-treated group. The results demonstrated that even though L-PWSI or D(+)Galactose treatment perturb learning and memory in Balb/c mice, both conditions together offer a worse outcome in the learning and memory ability in Balb/c mice.

### Spatial Working Memory and Episodic Memory Foundation Is Affected in the Y-Maze Test

The Y-maze experiment was performed due to the animals’ nature of showing a preference to explore novel locations rather than going back to a previously explored arm (Mo et al., 2013; Yang et al., 2019). In the Y-maze, the spontaneous test was performed to determine spatial working memory. Here, there are significant differences in the novel percentage [*F*_(3,24)_ = 15.28, *p* < 0.001, Figure 3A3] and total arm entries [*F*_(3,24)_ = 3.939, *p* < 0.05, Figure 3A2] but no significance in the novel arm entries [*F*_(3,24)_ = 2.767, *p* = 0.0637, Figure 3A1] among these groups. Compared to the control group, the L-PWSI group (*p* < 0.01) and the D(+)Galactose-treated group (*p* < 0.05) showed a significantly decreased novel percentage in the Y-maze. The novel percentage in the Y-maze was, however, worsened (in terms of reduction) when subjected to both L-PWSI and D(+)Galactose treatment compared to the D(+)Galactose group (*p* < 0.01) and the L-PWSI group (*p* < 0.05, Figure 3A3). Compared with the control, the D(+)Galactose group (*p* < 0.05, Figure 3A2) showed a significant increase in the total arm entries. Moreover, compared with the control, the L-PWSI group and the D(+)Galactose-treated group showed no significant changes in the number of novel arm entries (Figure 3A1).

maze new arm test was performed to detect episodic memory. After acquisition and 2 h consolidation, the retrieval of memory was tested (Figure 3B). Here, there were significant differences in the novel percentage [*F*_(3,24)_ = 25.31, *p* < 0.001, Figure 3B3], novel arm entries [*F*_(3,24)_ = 16.72, *p* < 0.001, Figure 3B1], and total arm entries [*F*_(3,24)_ = 16.31, *p* < 0.001, Figure 3B2] among the groups. When we compared the control with the D(+)Galactose group, a significant reduction was observed in the novel alternation whereas similar observation was made between D(+)Galactose group and D(+)Galactose + L-PWSI group were observed in the novel [*F*_(3,24)_ = 16.72, *p* < 0.001, Figure 3B1] and total alternations [*F*_(3,24)_ = 16.31, *p* < 0.001, Figure 3B2]. The percentage of novel arm entries also showed a significant reduction in the D(+)Galactose and L-PWSI groups when compared to the control group. Similarly, a significant reduction was overserved in the L-PWSI and D(+)Galactose-treated group when compared with the L-PWSI only and D(+)Galactose only groups [*F*_(3,24)_ = 25.31, *p* < 0.001, Figure 3B3].

Altogether, these results further support that L-PWSI and D(+)Galactose treatments affect the foundation and retrieval memory within the exploratory behavior. Therefore, spatial working memory, and episodic memory foundation, is altered in this group than in any of the conditions alone.

### Passive Avoidance Behaviors Were Worsened in the Long-Term Post-weaned Social Isolation and D(+)Galactose-Treated Group

The PAT is usually performed to access the passive avoidance behaviors exhibited by mice and this can be used to determine some form of memory in mice. Here, no significant differences were observed in the latency [*F*_(3,24)_ = 0.1936, *p* = 0.8997, Figure 4A], frequency [*F*_(3,24)_ = 0.6323, *p* = 0.6014, Figure 4B], and total time spent to (in) the darkroom [*F*_(3,24)_ = 0.4677, *p* = 0.7075, Figure 4C] on the first day of the trial. However, there are significant differences in the latency [*F*_(3,24)_ = 11.07, *p* < 0.01, Figure 4D], frequency [*F*_(3,24)_ = 11.32, *p* < 0.01, Figure 4E], and permanence time [*F*_(3,24)_ = 9.664, *p* < 0.01, Figure 4F] to (in) the darkroom among these groups on the second day. The L-PWSI group and the D(+)Galactose group exhibited significantly reduced latency to the darkroom compared to the control group. Moreover, the latency to the darkroom was markedly reduced in the group with the double condition (L-PWSI and D(+)Galactose treatment) when compared with the D(+)Galactose only group (*p* < 0.05, Figure 4D). The frequency to the darkroom was significantly increased in the L-PWSI and D(+)Galactose-treated group when compared with the L-PWSI only and the D(+)Galactose only groups (*p* < 0.05, Figure 4E). The total time spent in the darkroom (known as permanence time) also revealed a significant increase in the L-PWSI only and the D(+)Galactose only groups when compared with the control group (*p* < 0.05, Figure 4F). Herein, the double treatment group (i.e., L-PWSI and D(+)Galactose group) showed no significant increase in the permanence time in the darkroom compared with the individual treatment groups.

### Impaired Synaptic Functions in the Long-Term Post-weaned Social Isolation and D(+)Galactose-Treated Group

After observing the changes in the behavioral experiments, postsynaptic density protein, PSD-95, levels in hippocampus lysates were assessed in the groups after subjecting the mice to various treatments, and significant differences are shown in PSD-95 [*F*_(3,9)_ = 25.81, *p* < 0.001, Figures 5A,B] by two-way ANOVA. PSD-95 was significantly reduced in the D(+)Galactose only (*p* < 0.01), when the L-PWSI only (*p* < 0.001) was compared with the control. Moreover, the L-PWSI and D(+)Galactose-treated group showed a significant decrease when compared with the D(+)Galactose only group (*p* < 0.01).

Dendritic spines play vital roles in the formation and maintenance of emotional circuits and synaptic plasticity and cognition (Lin et al., 2012; Oey et al., 2015). The dendritic spine structure was determined using the Golgi apparatus to stain the hippocampus and identified a significant reduction of dendritic spines in the D(+)Galactose group and the L-PWSI group when compared with the control (*p* < 0.001), and a significant reduction in the L-PWSI and D(+)Galactose-treated group when compared with only L-PWSI or D(+)Galactose-treated groups (*p* < 0.01) as showed in Figures 5C,D [*F*_(3,9)_ = 56.25, *p* < 0.001]. Together with the decreased PSD-95 protein expression, these results suggest that synaptic plasticity would be deteriorated by either L-PWSI or D(+)Galactose treatment and is worsened when treated with both L-PWSI and D(+)Galactose.

### Autophagy and Cell Cycle Are Inhibited in D(+)Galactose and Long-Term Post-weaned Social Isolation-Treated Mice

In our earlier publication, we reported on inhibited autophagy activity when mice were isolated. Therefore, the expression of proteins associated with autophagy and cell senescence was determined. In determining the protein expression of p-mTOR, there was a significant difference among the groups [*F*_(3,9)_ = 20.86, *p* < 0.01, Figure 6A]. The relative protein expression of mTOR was not significant in the groups [*F*_(3,9)_ = 3.074, *p* = 0.0834, Figure 6B]. We detected the p-mTOR (Ser2448) and mTOR individually because the antibodies for these two are of the same species (rabbit) likewise p-ULK1 (Ser757) and ULK1. Specifically, there was a significant increase of p-mTOR in the D(+)Galactose and L-PWSI when compared to the control. The phosphorylated mTOR was significantly higher when the 2 conditions are present. A similar observation occurred in the expression of p-ULK1 and ULK1 [p-ULK1: *F*_(3,9)_ = 15.48, *p* < 0.01; ULK1: *F*_(3,9)_ = 1.828, *p* = 0.2122, Figures 6C,D]. The activation of p-mTOR inhibits autophagy, while the Beclin1 promotes autophagy (Cordaro et al., 2016). Beclin1 was, therefore, detected as well and found a significant decrease in D(+)Galactose only-treated and L-PWSI only-treated groups when compared with the control, whereas no significant between D(+)Galactose and L-PWSI double-treated group and D(+)Galactose or L-PWSI only-treated group [*F*_(3,9)_ = 28.17, *p* < 0.001, Figure 6F]. p62, as an autophagy substrate, is degraded during autophagy activation (Lau et al., 2013). The p62 protein expression was significantly upregulated in the D(+)Galactose (*p* < 0.05) and L-PWSI-treated (*p* < 0.01) groups compared to the control group. It was also observed that the expression was significantly upregulated in the D(+)Galactose and L-PWSI double-treated (*p* < 0.01) group relative to the D(+)Galactose treatment only [*F*_(3,9)_ = 21.27, *p* < 0.01, Figure 6E]. The decrease in Beclin1 and accumulation of p62 are indicative that autophagy is inhibited. Here, this study is the first to report on this relationship between autophagy with aging and isolation. All these together support the fact that D(+)Galactose impairs autophagy.

To verify that the aging model was indeed established, cell cycle markers [p16, cyclin D1, and proliferating cell nuclear antigen (PCNA)] were determined. The expressions of p16 [*F*_(3,9)_ = 21.42, *p* < 0.01, Figure 6G] and cyclin D1 [*F*_(3,9)_ = 32.82, *p* < 0.01, Figure 6H] were found to be significantly increased in the D(+)Galactose only and L-PWSI only groups but were worse when the two conditions exist. PCNA expression was significantly decreased in the D(+)Galactose only and L-PWSI only groups [*F*_(3,9)_ = 16.68, *p* < 0.01, Figure 6I]. Furthermore, antioxidant molecules, Nuclear factor erythroid 2-related factor 2 (Nrf2), Heme Oxygenase-1 (HO-1), and Superoxide dismutase (SOD) expressions were in turn determined and the results revealed significant decreases when the D(+)Galactose only and L-PWSI only treated when compared with the control and worsened when both-treated as shown in the Supplementary Figure 1. The antioxidant activity is also associated with age and cell senescence. Altogether, these suggest that aging was established and that the phenomenon leading to cognitive decline in these model groups could be linked to autophagy and cell cycle processes.

## Discussion

The prevalence of social isolation or loneliness has been reported to be high in the older population as reviewed by Ong et al. (2016). In their review, they provided enough evidence that supported the conclusion that social isolation or loneliness at an older age is high enough to warrant an intervention. The proposal on the need to clarify the brain mechanisms underlying the association between social isolation (loneliness), old age, and cognitive decline and also consider the extent to which such decline can be reversible through some interventions is important (Ong et al., 2016). It is in light of this that the current study was designed to provide preliminary data to initiate interest in this area of research. This current study was designed to determine the combined effect of the social isolation model and aging on cognition in mice. The present data are approved by some research, as Green et al. (2008) found that there is a cross-association between social network and cognition and functional status in older adults.

The results obtained indicate the damaged kinds of memories foundation in the individual treatment with L-PWSI or D(+)Galactose as well the L-PWSI and D(+)Galactose together. It should be noted that the L-PWSI worsened the D(+)Galactose-induced cognitive and aging phenotype, as impaired learning, synaptic protein deficiencies, and inhibited autophagy and cell cycle [D(+)Galactose vs. L-PWSI + D(+)Galactose]. While the D(+)Galactose drug showed less impact on L-PWSI-induced cognition- and aging-related index changes [L-PWSI vs. L-PWSI + D(+)Galactose]. This proves the protective effects of social relationships against the aging process (Uchino, 2006).

In our previous study, the L-PWSI model tends to show impaired cognitive function and decreased PSD-95 in mice models (Wang et al., 2019). The decreased release of PSD-95 from the postsynaptic membranes could later impact postsynaptic restructuring to eventually result in dysfunction of synaptic plasticity (Wang et al., 2019). This observation is similar to the results of this study. Moreover, the loss of the dendritic spines proved the reduced synaptic function and consequently results in cognitive deficits. Our previous study was a maiden study to propose that autophagy inhibition damages synapses and affects cognition.

Inhibition of autophagy activity by isolation and drug-induced aging is reported in the current study. Autophagy has already been studied for its anti-aging effects (Rubinsztein et al., 2011). The p-mTOR, p-ULK1 (Ser757), p62 proteins typically increase with age (Vilchez et al., 2014; Yuan et al., 2018). As a major inhibitor of autophagy, high mTOR activity prevents ULK1 activation by phosphorylating ULK1 at Ser 757 and subsequently suppressing autophagy (Rubinsztein et al., 2011). L-PWSI worsened the D(+)Galactose-induced hippocampal autophagic inhibition *via* activating the p-mTOR and phosphorylating ULK1 at Ser 757 to inhibit its activation. The inhibited autophagy is further confirmed by a decrease of Beclin1 and accumulation of p62 (Wang et al., 2018). Basal autophagy positively regulates synaptic development and by extension improves cognitive performance (Shen and Ganetzky, 2009). Decreased levels of autophagy may lead to loss of protective function of neurons resulting in cognitive impairment (Gao et al., 2015; Li et al., 2017).

As depicted in the study of Coryell et al., the conclusion is that autophagy regulates the degradation of p16 (cell senescence marker) (Coryell et al., 2020) and has been reported to be increased in aged mice and therefore, inhibition of p16 may reverse aging progress (Boquoi et al., 2015; Ma et al., 2018). Accumulated p16, collectively known as cyclin-dependent kinase (CDK) inhibitor with the most prominent role in senescence cell accumulation during aging, can drive cellular senescence (Di Micco et al., 2021). Cyclins D1 belonging to the family of CDKs/cyclin complexes, which promote the transition from G- to S-phase of the cell cycle, is closely linked with cell senescence (Choi and Anders, 2014; Tyson and Novák, 2015). Cyclin D1 activates CDK4/6 to form cyclin D1-CDK4/6 complexes, which would accumulate during the G1 phase until DNA replication, then cyclin D1 is exported to the cytoplasm where it is degraded by autophagy (Tchakarska and Sola, 2020). In the present research, the autophagy inhibition attenuated the degradation of p16 and cyclin D1. Furthermore, the accumulation of cyclin D1 inhibits cell cycle progression by forming a complex with the PCNA and keeping PCNA inactivated, as revealed by the decreased PCNA in our current study (Xiong et al., 1992). The expression of PCNA can reflect the proliferation of cells (Zhao et al., 2014). These results are corroborated by our findings that cyclin D1 increased in the hippocampus when mice were subjected to D(+)Galactose or L-PWSI treatments whereas PCNA decreased in these mice compared to the control. This demonstrates that D(+)Galactose or L-PWSI treatment would arrest the cell cycle and can be concluded to some extent that L-PWSI treatment worsens cells cycle arrest in the mice administered with D(+ Galactose). Senescence is believed to be a permanent condition of cell cycle arrest (Lee and Lee, 2019). In the light of these, we propose that D(+)Galactose or L-PWSI treatment would induce cell senescence in mice *via* disturbing the autophagy and that L-PWSI treatment accelerated the D(+)Galactose-induced senescence by worsening the autophagy which is hampered by D(+)Galactose administration. Apart from that, aberrant DNA replication and DNA damage accumulation are important characteristics of senescence as well (Di Micco et al., 2006). In the future, autophagy-related inhibition will be done to confirm the exact role of autophagy in disrupting senescence.

Research by Fulopet et al., and Hiebert et al., reported that the level of Nrf2 decreases with age and silencing of the Nrf2 gene is associated with the induction of premature senescence (Fulop et al., 2018; Hiebert et al., 2018). Nrf2 can bind to the conserved antioxidant response element (ARE) of a series of antioxidative targets, such as HO-1 (Jadeja et al., 2020). These studies together with these current results support the onset and change in senescence in each group. However, how autophagy is influenced by isolation in detail and the direct evidence to change aging and cell senescence are not sufficient and therefore warrant more research.

## Conclusion

This study reports on how induced social isolation and aging could cause changes in the brain resulting in cognitive deficits or impairments. Autophagy in the D(+)Galactose treatment alone and the double treatment was done to elucidate the possible molecular linkage of these two phenomena (i.e., aging and social isolation). Furthermore, a clue about how isolation affects autophagy and the cell cycle is revealed in this study and how both are vital for aging. The inevitable physiological state of aging and the increasing spate of social isolation in the old age group in recent times can exacerbate the case of impaired learning and memory, which could increase the global burden of disease in the future.

## Data Availability Statement

The raw data supporting the conclusions of this article will be made available by the authors, without undue reservation.

## Ethics Statement

The animal study was reviewed and approved by Animal Studies Committee, Dalian Medical University.

## Author Contributions

SL, BW, and J-YY contributed to the conception and design of the project. BW, MN, YW, MX, and J-cL contributed to the conduct of the experiments and analysis of data. BW and MN wrote the manuscript. All authors contributed to the article and approved the submitted version.

## Conflict of Interest

The authors declare that the research was conducted in the absence of any commercial or financial relationships that could be construed as a potential conflict of interest.

## Publisher’s Note

All claims expressed in this article are solely those of the authors and do not necessarily represent those of their affiliated organizations, or those of the publisher, the editors and the reviewers. Any product that may be evaluated in this article, or claim that may be made by its manufacturer, is not guaranteed or endorsed by the publisher.
