# Supplementary material for: Long-Term Social Isolation-Induced Autophagy Inhibition and Cell Senescence Aggravate Cognitive Impairment in D(+)Galactose-Treated Male Mice
Source: Front Aging Neurosci. 2022 Mar 24;14:777700. doi: 10.3389/fnagi.2022.777700 (PMC8988191; doi:10.3389/fnagi.2022.777700)
Supplement: Supplementary file 1 [file Image_1.pdf]

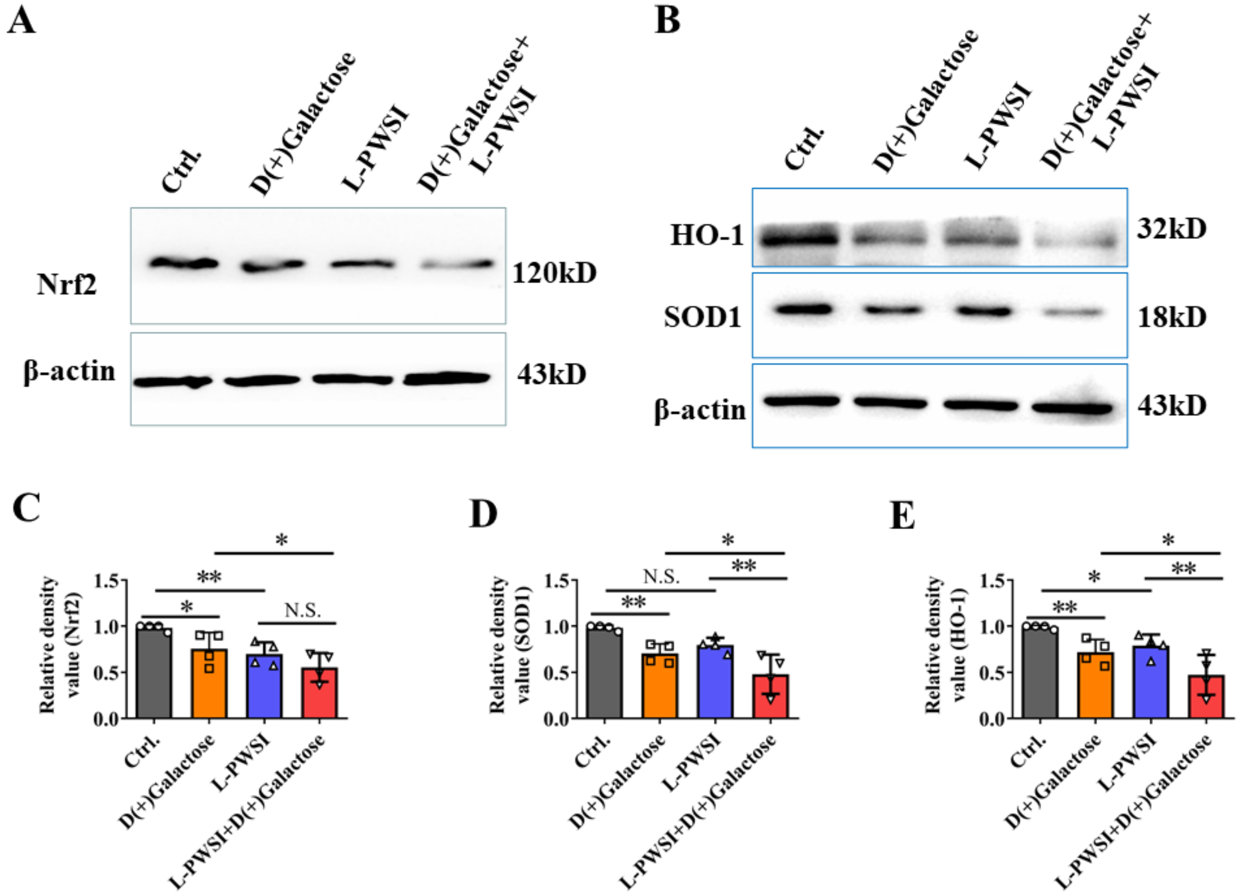

Figure Legend

Fig. S1

Treated with L-PWSI and D(+) Galactose altered anti-inflammation-related molecules.

Representative micrographs **(A, B)** of Western blot (protein expression) with the densitometry analysis of Nrf2 **(C)**SOD1**(D),** and HO-1**(E)** in the hippocampus. The grouping of gels/blots is cropped from different parts of the same gel.Each data column represents the mean ± SEM obtained from 4 brain samples. * represents $p< 0.05$; ** represents $p< 0.01$; *** represents $p< 0.001$; N.S. represents no significance.
